# Molecular Pathways Driving Corneal Neovascularization in Herpes Simplex Keratitis

**DOI:** 10.3390/pathogens15020186

**Published:** 2026-02-07

**Authors:** Soromidayo Akinsiku, Deepak Shukla

**Affiliations:** 1 Department of Ophthalmology and Visual Science, College of Medicine, University of Illinois Chicago, Chicago, IL 60612, USA; sakins2@uic.edu; 2 Department of Microbiology and Immunology, College of Medicine, University of Illinois Chicago, Chicago, IL 60612, USA

**Keywords:** herpes simplex virus type 1, herpetic stromal keratitis, corneal neovascularization, angiogenesis, VEGFA, JAK/STAT signaling

## Abstract

Herpes simplex keratitis (HSK) is classically described as an immunopathological disease driven by recurrent herpes simplex virus type 1 (HSV-1) infection and chronic inflammation. So far, immune-mediated tissue damage has not fully explained the molecular mechanisms governing disease progression toward corneal neovascularization (CNV), a major cause of corneal blindness and vision loss worldwide. Increasing evidence indicates that CNV results from complex interactions that extend beyond leukocyte-driven inflammation, as the host cell machinery, including key pathways and molecular markers, is hijacked by the invading virus to establish and perpetuate replication and lifelong latency. These host–cell interactions regulate angiogenic imbalance, vascular privilege, and tissue remodeling, which collectively promote pathological vascular invasion. This review re-examines HSK by focusing on molecular mechanistic pathways and drivers that regulate disease progression towards CNV, upstream of immune response drivers. Specifically, we discuss the roles of endothelial growth factors, matrix metalloproteinases, Heparanase, and Syndecan-1 signaling, as well as microRNA-mediated regulation, and key signaling axes, including JAK2/STAT3, PI3K/AKT/mTOR, and hypoxia signaling. By integrating these pathways and molecular markers, we propose an updated mechanistic framework, including a conceptual model for the underexplored role of heparanase, and identify pathway-level targets with potential therapeutic relevance for HSK-associated CNV.

## 1. Introduction

Herpes simplex virus (HSV) comprises two subtypes, HSV-1 and HSV-2, primarily associated with ocular and genital infections, respectively [1,2]. HSV-1 establishes lifelong latency in the host with the capacity for periodic reactivation, a feature that underlies recurrent corneal disease collectively referred to as herpes simplex keratitis (HSK) [3]. HSK is a clinically significant, potentially blinding disease resulting from recurrent corneal infection [3]. Studies on the global burden of HSV-1 and HSK have estimated that approximately 4 billion people are infected with HSV-1, with 1.5 million cases of HSK worldwide, and about 500,000 cases in the United States alone [1,4,5,6,7]. HSK is also a leading cause of visual impairment, although the disease is predominantly unilateral, with approximately 40,000 cases of unilateral blindness annually; bilateral involvement has been reported in 1.2 to 12% of cases, especially in younger adults, highlighting the clinical heterogeneity and potential severity of HSK [3,5,6,8,9].

HSV-1 infection affects the conjunctiva, retina, and iris; however, corneal infection and subsequent disease account for the most severe and chronic infectious cause of vision impairment [3,10]. HSK pathogenesis presents as stroma, endothelial, epithelial, and uveitis keratitis, and among these presentations, the recurrent and stromal forms of keratitis are most strongly associated with clinical manifestations such as corneal scarring, opacity, and edema, CNV, and reduced visual acuity [3,10,11]. Following primary HSV-1 infection, the cornea mounts a robust inflammatory response characterized by the recruitment of innate immune cells, including neutrophils, macrophages, and innate-like lymphocyte populations, along with the release of pro-inflammatory chemokines and cytokines that amplify immune cell infiltration [12,13,14,15]. At later clinical stages, stromal lesions are dominated by CD4^+^ T cells, particularly Th1 and Th17 subsets, which outnumber CD8^+^ T cells and serve as key mediators of immunopathology [16,17,18]. These effector T cells produce high levels of inflammatory cytokines, including IFN-γ, IL-17, TNF-α, and IL-1β, which act on resident corneal epithelial, stromal, and vascular endothelial cells to induce pro-angiogenic mediators, most notably VEGFA [19,20,21]. Consequently, tissue damage in HSK is generally considered immune-mediated rather than a direct consequence of active viral replication, highlighting the central role of host inflammatory responses in disease pathogenesis. However, despite extensive characterization of immune-driven mechanisms, limitations in current therapies and accumulating evidence of intrinsic host cell-viral interactions suggest that additional, non-redundant regulatory layers contribute to disease progression and pathological angiogenesis [16,21,22,23,24,25,26].

Although immune-regulated responses constitute an important component of HSK-induced corneal neovascularization (CNV), this pathological process is not exclusively immune-driven. In the early phase of infection, i.e., HSV-1-infected corneal epithelial cells, stromal fibroblasts respond to HSV-1 infection and associated inflammatory cues by engaging intracellular signaling programs and molecular pathways that influence the balance between pro- and anti-angiogenic signals, occurring prior to substantial leukocyte infiltration and independent of immune-derived cytokine cues [21,27,28,29]. Consistent with this, HSV-1 infection can directly increase VEGFA output from infected corneal cells, and disease progression reflects a dynamic interplay in which host cell–intrinsic signaling seeds an angiogenic program that is subsequently amplified and reshaped by immune-derived cytokines and inflammatory proteases [21,27]. Accordingly, dysregulation of these host–cell pathway machineries, such as JAK/STAT and PI3K-AKT, are best viewed as dual-use module: functioning as (i) immune-independent, cell-intrinsic triggers early in infection, and (ii) immune-modulatory amplifiers once cytokine-driven immune networks intensify by promoting loss of corneal angiogenic and immune privilege and vascular invasion [24,29,30,31,32]. Therefore, understanding how host–cell intrinsic mechanisms contribute to CNV pathogenesis is essential for identifying novel therapeutic targets addressing the molecular drivers of pathological angiogenesis in HSK. In this review, we discuss the molecular and cellular mechanisms that drive CNV in the context of HSK. We first highlight the mechanism underlying corneal angiogenic privilege, outline the pathological features of HSK-induced CNV, and highlight the distinct contributions of corneal resident cells and infiltrating immune populations to the initiation and progression of angiogenesis. We then examine key inflammatory and growth factor-mediated signaling pathways implicated in HSK-associated CNV, with a particular emphasis on host signaling networks, including JAK/STAT, PI3K/AKT, Notch, ROCK, and hypoxia-HIF-1α signaling. Throughout these sections, we identify key findings from non-infectious CNV models and explain how they can be extrapolated to HSK-induced CNV investigations. We also highlight points of pathway crosstalk and convergence on VEGFA-dependent endothelial activation. We further discuss the emerging regulatory roles of microRNAs and extracellular matrix-associated modulators such as heparanase, syndecan-1, and osteopontin in shaping angiogenic and immune signaling networks within the cornea. In addition, we summarize established findings into integrated mechanistic pathway models and propose a conceptual framework for the largely unexplored role of heparanase in HSK-induced CNV. Finally, we integrate these mechanistic insights to identify unresolved questions and emerging therapeutic targets for HSK-induced CNV.

## 2. Angiogenic Privilege, Angiogenesis, and CNV

### 2.1. Cornea and Angiogenic Privilege

The cornea is avascular, allowing unimpeded light to reach the retina for clear vision. This avascularity is actively maintained through angiogenic privilege, which relies on dominant anti-angiogenic mechanisms rather than the absence of pro-angiogenic factors [33,34]. Angiogenic privilege is regulated by the delicate balance of molecules traditionally categorized as pro- and anti-angiogenic, although some molecules exhibit both properties depending on the physiological context [35]. Key pro-angiogenic factors implicated in CNV include VEGFA, matrix metalloproteinases (MMPs), angiopoietins, platelet-derived growth factor (PDGF), hypoxia-inducible factors (HIFs), inflammatory cytokines and chemokines, and extracellular matrix-associated regulators, whereas anti-angiogenic proteins include soluble VEGF receptor-1 (sVEGFR-1), pigment epithelium–derived factor (PEDF), thrombospondins, tissue inhibitors of metalloproteinases (TIMPs), endostatin and anatomical and molecular barriers at the limbus that restrict vascular ingrowth [35,36]. Together, these mechanisms preserve corneal transparency under homeostatic conditions.

Angiogenic privilege can be lost as a result of infection, injury, or trauma [37]. HSV-1 infection disrupts these safeguards by shifting the balance toward bioavailable, pro-angiogenic signaling. Infected corneal epithelial cells directly upregulate VEGF-A expression, while inflammatory proteases and matrix remodeling enzymes degrade anti-angiogenic restraints, including sVEGFR-1, thereby unmasking VEGFA activity at the limbal vasculature [28,30]. This breakdown of corneal angiogenic privilege lowers the threshold for endothelial activation and vessel ingrowth, creating a permissive microenvironment in which immune-derived cytokines and infiltrating leukocytes further amplify and sustain pathological neovascularization [38]. Therefore, HSK-associated CNV is regarded as a consequence of failed angiogenic privilege, underscoring why VEGF-A can be present in the healthy cornea without inducing vascularization, yet rapidly promotes CNV following viral infection.

### 2.2. Angiogenesis Versus CNV

Angiogenesis is the formation of new blood vessels from pre-existing vasculature. It is a complex mechanism chiefly mediated by VEGFA, and regulated by pro-inflammatory factors, hypoxia-inducible factors, and a host of signaling pathways [39,40]. This process involves endothelial cell activation, proliferation, degradation of the basement and ECM, and formation of tubular networks, and this occurs in both physiological and pathological conditions [41,42,43]. On the other hand, CNV is a critical pathological event resulting from the host response in HSK and other inflammatory and injury-related conditions, leading to the formation of blood vessels in the cornea from the surrounding limbal vasculature. In general, neovascularization is essential and helpful for the clearance of infection, in wound healing and tissue repair; however, in the cornea where avascularity is essential and maintained for a healthy vision, the incursion of blood vessels in the cornea leads to the disruption of the highly organized architecture of the corneal stroma rendering the invading vessels structurally aberrant and functionally detrimental, leading to loss of transparency and vision impairment even at low vessel densities [33,35]. While physiological angiogenesis is governed by a balance between pro- and anti-angiogenic factors, the cornea is uniquely maintained in an actively avascular state through dominant anti-angiogenic mechanisms. Consequently, CNV requires a profound disruption of anti-angiogenic signaling and an activation of pro-angiogenic signaling pathways [42]. Therefore, what makes CNV unique is its role in the loss of ocular immune privilege and inflammation-driven vascular remodeling that leads to vision loss [44,45]. Table 1 summarizes the key features that differentiate physiological angiogenesis from CNV.

### 2.3. CNV Models

CNV is a vision-threatening clinical manifestation of various pathological assaults, such as injury, inflammation, limbal barrier loss, hypoxia, and infection [46]. To investigate the mechanisms underlying CNV, a range of experimental models have been developed to induce pathological angiogenesis in the cornea. These include fungal infection, chemical injury (alkaline or silver nitrate burns), corneal suturing, immunogen-induced inflammation, tumor cell implantation, and HSV-1 infection [47,48]. Although comparative studies remain limited, available evidence indicates that distinct etiological models can produce differences in vascular architecture, stromal remodeling, and inflammatory signaling, and the extent to which this affects pathogenesis and therapeutic interventions remains to be explored. This review will focus on HSK-induced CNV.

### 2.4. Cell-Type Specific Roles in CNV Pathogenesis

The cornea is divided into 5 layers: corneal epithelium, Basement/Descemet Membrane, Bowman’s layer, stroma, and corneal endothelium, and each of these plays an important role in mitigating against infection [49]. In HSK-induced CNV, the primary targets of HSV-1 infection are the corneal epithelium and stroma, giving rise to a pathology that traverses the corneal layers in 3 types of CNV: superficial, stromal, and deep neovascularization [50]. Based on available evidence, HSK-induced CNV arises from coordinated responses among resident corneal cells, limbal vascular endothelium, infiltrating leukocytes, and vascular support cells. Corneal epithelial cells are the primary site of HSV-1 infection and the initial trigger of angiogenic signaling. Viral replication and epithelial injury induce the release of pro-inflammatory and pro-angiogenic mediators, including IL-1, IL-6, VEGFA, and angiopoietin-2 (Ang2), as well as MMPs that degrade the ECM, initiating vascular activation [21,46,51]. Activated corneal stromal keratocytes actively remodel the extracellular matrix to create permissive stromal spaces and secrete angiogenic cues that guide endothelial invasion [52]. Limbal vascular endothelial cells (VECs) respond to epithelial-derived signals and inflammatory cytokines by activating VEGFR2-dependent signaling pathways, leading to endothelial activation and proliferation at the limbal arcade [46,47,49].

Also, innate immune cells, particularly γδ-T cells, neutrophils, and macrophages, are rapidly recruited to the infected cornea. These cells produce VEGFA, IL-17, and TGF-β, thereby further amplifying endothelial activation and lowering the angiogenic threshold [19,28,30,42,51]. Pericytes contribute to later stages of vascular remodeling in CNV. In alkali-induced CNV models, increased pericyte association with neovessels correlates with altered vascular permeability and vessel maturation status, suggesting a context-dependent regulation of barrier function during pathological angiogenesis [53]. This behavior is consistent with the established roles of pericytes in regulating vascular stability and permeability in other barrier tissues, including the blood-retinal barrier and blood–brain barrier, where pericyte dysfunction is associated with increased leakage rather than vessel stabilization [53,54,55]. Given the multicellular nature of HSK-induced CNV, Table 2 outlines the major corneal resident and infiltrating cell populations involved in disease progression, together with their dominant angiogenic outputs and functional contributions.

## 3. Overview of Key Host–Cell Molecular Pathway Drivers in the Pathogenesis of HSK-Induced CNV

### 3.1. VEGFA and sVEGFR-1 Balance as an Important Index of CNV

VEGF-A is the primary driver of CNV in HSK. Under normal physiological conditions, it functions in vasculogenesis, capillary permeability, endothelial cell proliferation, and migration [43,56]. Although present in a healthy cornea, VEGFA remains bound to decoy cell surface receptor with low kinase activity, present as soluble and transmembrane form as s-VEGFR1 and full-length VEGFR1 (FLT-1), respectively, inhibiting its ability to interact with kinase-active VEGF receptor-2 (KDR) on the vascular endothelium; thereby maintaining avascularity via homeostatic balance [28,33,35]. During primary HSV-1 infection, the virus establishes lifelong latency by entering the sensory trigeminal ganglion [10,14]. Reactivation of HSV-1 occurs in the event of immunosuppression or UV-activation, triggering CNV by upregulating *VEGFA* gene expression, thereby disrupting the angiogenic balance. Thus, CNV does not arise solely from increased *VEGFA* expression, but rather from a disruption in the homeostatic balance between pro-angiogenic VEGFA and its endogenous decoy receptor sVEGFR-1, allowing unrestrained VEGFR-2 activation. Importantly, this imbalance is further exacerbated by proteolytic degradation of soluble VEGFR-1 by matrix metalloproteinases, such as MMP-7 [53], a mechanism discussed in subsequent sections.

The pathogenic development of CNV begins with the release of VEGFA by multiple cell types, including epithelial cells, stromal keratocytes, immune cells, vascular endothelial cells, and pericytes [21,42]. The binding of VEGFA to VEGFR2 on peri-limbal vascular structures activates them by autophosphorylation and dimerization, initiating VEC activation and leading to the migration of vascular tissues into the stroma as they produce new vessels towards the site of VEGFA release [57].

The major source of VEGFA in HSV-1 is a matter of debate. A study has identified infected epithelial cells as the major sources of VEGFA and has also demonstrated that ICP4, an HSV-1 viral transcription factor, binds to the *VEGFA* promoter, as is the case with immediate-early viral genes, to upregulate its expression in the corneal epithelium [21]. Other studies have highlighted that IL-6 produced by infected cells [16,42] and IL-17 produced by infiltrating γδ-T cells during adaptive immune response stimulate VEGFA expression in neighboring cells [19,30]. In addition, the release of pro-inflammatory factors, such as neutrophils (PMNs) and macrophages, is a significant source of VEGFA and proteases, which downregulate sVEGFR-1, freeing up more VEGFA to initiate angiogenesis [28]. It is therefore probable that temporal and cell-type-specific differences in VEGFA sources during infection contribute to the biphasic pattern of VEGFA expression observed in HSK-associated CNV [42].

**Figure 1:**
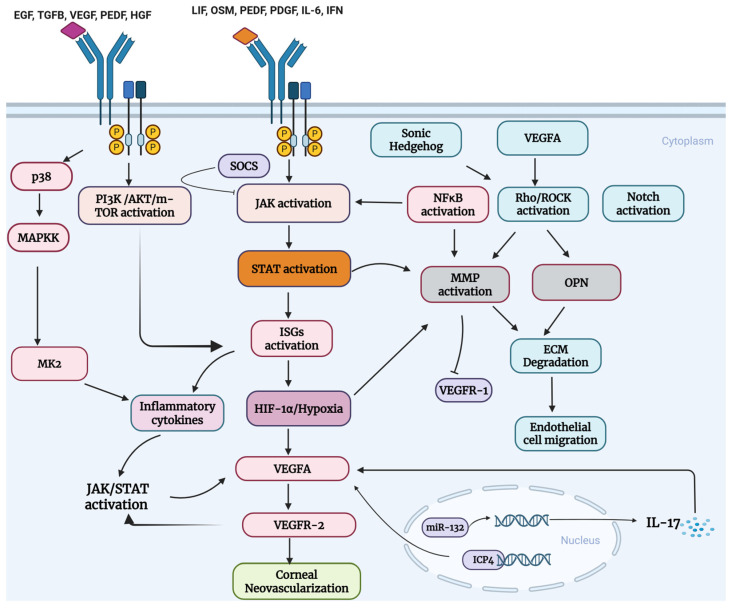
A systems-level overview of signaling convergence in HSK-associated CNV. Activation of cytokine and growth factor receptors triggers JAK/STAT and PI3K/AKT/mTOR signaling cascades, which intersect with MAPK, NF-κB, Rho/ROCK, and Notch pathways to regulate inflammatory responses, extracellular matrix remodeling, and endothelial cell behavior. JAK/STAT signaling induces interferon-stimulated genes and inflammatory cytokines, which may promote hypoxia-associated responses and VEGFA expression. Concurrently, PI3K/AKT signaling integrates inputs from VEGFR2 and other receptor tyrosine kinases to support endothelial proliferation and survival. NF-κB- and Rho/ROCK-dependent activation of MMPs facilitates ECM degradation and endothelial migration, enabling pathological vascular sprouting. Viral and immune-derived factors, including HSV-1 regulatory proteins and IL-17, further modulate angiogenic gene expression. Created in BioRender. Akinsiku, S. (2026) https://BioRender.com/sj0erfk. Accessed 27 January 2026.

Although HSV-1 actively induces VEGFA expression, and VEGFA is a major driver of the CNV that worsens HSK pathology, whether its upregulation confers a direct replication advantage to HSV-1 in the cornea remains less clearly defined. While the role of VEGFA as the major driver of CNV has been established in the literature [58], the contribution of various molecules and upstream inflammatory, cell-survival, growth, proliferation, migration, and immune response pathways regulating VEGFA, which become hijacked and/or are engaged by invading viral machinery to perpetuate its survival, remains largely underexplored. These pathways include PI3K-AKT, JAK/STAT, Rho-ROCK, and Notch signaling. To integrate the diverse host signaling pathways implicated in HSK-associated CNV, Figure 1 provides a systems-level overview of signaling pathways and convergence in HSK-induced CNV. We will discuss details in subsequent sections.

### 3.2. Matrix Metalloproteinases (MMPs) and TIMPs Ratio in CNV

As highlighted above, in the pathogenesis of CNV, activated perilimbal vascular structures release zinc-dependent proteolytic enzymes, such as MMP-2, -7, and -9, degrade the surrounding extracellular matrices and basement membrane, and release pro-angiogenic proteins, thereby supporting their uninhibited incursion into the corneal stroma [59]. The role of MMPs in enhancing the activation of vascular endothelial migration and invasion in cancer has also been established [60,61]. In CNV, recruited neutrophils release MMPs, particularly MMP-9, which contribute to extracellular matrix degradation and tissue remodeling during CNV, further perpetuating the homeostatic imbalance in angiogenic factor levels [28,36,42]. These MMPs act on most of the components of the ECM, including gelatinases A/B, collagenases 1/3, stromelysin-1, and matrilysin. MMP-1, -2, -3, -7, -9, -10, -13, and -14 are expressed in corneal tissues and are maintained under homeostatic control by tissue inhibitors of metalloproteinases (TIMPs); however, excessive MMP expression can disrupt this balance, promoting extracellular matrix degradation and facilitating CNV. TIMPs exert inhibitory functions on MMPs by binding them in a 1:1 ratio, thereby maintaining ECM integrity. They act as active inhibitors of MMPs by binding to them in both the pro-enzyme and activated states [62]. However, in some contexts, MMPs can exhibit antiangiogenic properties by cleaving the pro-enzymes of antiangiogenic factors like angiostatin and endostatin [63]. MMP-9 has been demonstrated to play a key role in HSK-induced CNV, as evidenced by the reduction in CNV severity observed in MMP-9 knockout mice and with the inhibition of MMP-9 by TIMPs [64].

### 3.3. JAK-STAT Signaling Pathway in HSV-1 Infection

The JAK-STAT pathway mediates several cellular responses, including proliferation, differentiation, migration, metabolism, immune-inflammatory responses, apoptosis, and cell survival, depending on the upstream cytokine signal [65]. It is composed of four-membered non-receptor tyrosine kinases JAK family (JAK1, JAK2, JAK3 and TYK2) which are found attached to the intracellular domains of cytokine receptors, and the seven transcriptional factors STAT family (STAT1, STAT2, STAT3, STAT4, STAT5A, STAT5B, STAT6) that are connected to the JAK family in a process that translate signaling to downstream activation of effectors as well as gene transcription.

Extracellular ligand binding, such as IL-6 to the IL-6 receptor, leads to pathway activation and thus the autophosphorylation of clustered JAKs at the base of the receptors. This secondary activation of receptors results in downstream activation of STATs by their docking to the bases of phosphorylated receptors, which are activated by JAKs. Phosphorylated STATs then form a broad range of homodimers and heterodimers, which then translocate into the nucleus to initiate extensive transcriptional gene regulation that mediates cellular processes [31,65]. In the cornea, known activators of this pathway include cytokines like LIF (Leukemia Inhibitory Factor) and CNTF (Ciliary Neurotrophic Factor), oncostatin M (OSM), and IL-6; growth factors such as transforming growth factor-β (TGF-β), platelet-derived growth factor (PDGF), pigment epithelium-derived factor (PEDF), and neuregulin-1 (NR-1), and negative regulators include miRNAs and Suppressors of Cytokine signaling (SOCS1-3), PIAS (protein inhibitors of activated STAT), and PTP (protein tyrosine phosphatases) [31,65,66,67,68,69].

Following HSV-1 infection, interferon (IFN) and other cytokines are released by components of the innate immune system, triggering an antiviral state in tissues. IFN then binds to its receptor, IFNα receptor, to activate the JAK-STAT pathway, leading to downstream activation of IFN-stimulated genes (ISGs) and pro-inflammatory cytokines, which rapidly clear the invading virus and stimulate the adaptive immune system [70,71]. Because this signaling pathway is conserved, the HSV-1 deploys various mechanisms to subvert it, such as the expression of UL36USP, a ubiquitin-specific protease that antagonizes type I interferon signaling by disrupting JAK–STAT activation, and UL42, which suppresses ISGF3-dependent transcription of interferon-stimulated genes, thereby reducing antiviral ISG expression [72,73]. HSV-1 also expresses protein kinase UL13 to upregulate SOCS1 and SOCS3, naturally occurring negative regulators of the JAK-STAT pathway [74]. Therefore, this suggests that downregulation of this pathway is essential for HSV-1 survival, making JAK-STAT signaling a crucial target in HSK-induced CNV therapeutics. Interestingly, components of the JAK-STAT pathway have been described to possess both pro- and anti-viral functions, depending on the context and the virus, although the exact mechanisms underlying these functions have not yet been established. Studies have established antiviral roles for STAT1 and STAT3, as evidenced by decreased viral replication in dendritic cells and macrophages from STAT1-deficient mice, and impaired interferon and ISG production in bone marrow-derived macrophages from STAT3-deficient mice following HSV-1 infection [75,76]. In the context of HSV-1 infection, the JAK1/2 inhibitor ruxolitinib has been classified as proviral in pathway-based analyses due to its suppression of IFN-mediated JAK-STAT signaling, a central antiviral axis actively targeted by HSV-1 [31]. In contrast, the kinase inhibitor AT-9238 has been associated with a significant reduction in neuronal HSV-1 infection in cell-based screening studies and has since advanced to Phase III clinical trials for cancer treatment [77]. The seeming duality in the therapeutic response to JAK inhibition highlights the complexity of the viral-JAK-STAT pathway interaction and explains the limitations of our current understanding of the molecular mechanisms that regulate CNV.

### 3.4. JAK-STAT-VEGFA Signaling

Studies have demonstrated that this pathway plays a crucial role in regulating angiogenesis. Notably, the *VEGFA* gene promoter contains sequences that serve as STAT3 binding sites upon its nuclear translocation, establishing STAT3 as a key regulator of *VEGFA*. Consequently, for example, the overexpression of STAT3 has been found to increase *VEGFA* expression in cardiac muscles, thereby enhancing the formation of microvascular vessels required for cardiac remodeling [69]. In one study investigating the role of JAK-STAT signaling in angiogenesis, a 3D-gel coculture of endothelial cells and adipose-derived stromal cells demonstrated increased angiogenesis when treated with pathway activators, resulting in the upregulation of *VEGFA* and other pro-angiogenic factors [78]. On the other hand, STAT activation in aortic endothelial cells has also been shown to be mediated by VEGFA-VEGFR2 signaling [41], suggesting a positive feedback signaling between angiogenic and inflammatory pathways. Furthermore, its role in the regulation of corneal metabolism, inflammation, CNV, and corneal fibrosis has also been highlighted in the literature. Studies have established the role of IL-6-activated JAK/STAT3 in the pathogenesis of corneal alkali burn-induced CNV via *VEGFA* upregulation, with observed attenuation of CNV severity with inhibitors of this pathway [79,80,81,82,83]. However, while the crucial role of this pathway has been established in cardiovascular diseases, cancer, inflammation, and tumor angiogenesis, as well as some corneal diseases [41,65,84], its role in HSK-induced CNV remains underexplored.

### 3.5. JAK-STAT-MMPs Signaling

MMPs are regulated transcriptionally as targets of upstream cellular signaling in response to cytokine-induced stimulation. Such upstream pathways include nuclear factor kappa B (NF-κB), the mitogen-activated protein kinases (MAPK), and STAT. MMP promoters consist of response elements for transcription factors such as STAT, activator proteins AP-1 and -2, and NF-κB [85]. The role of STAT-MMPs signaling is evident in several studies that show a correlation between STAT inhibition and MMPs downregulation. Specifically, in an endothelial-stromal cell co-culture model, treatment with the STAT inhibitor Stattic has been reported to downregulate MMP-2, MMP-9, VEGFA, and other pro-angiogenic factors [78]. In a separate study, treatment with a cyclic sesquiterpene, Zerumbone, in an alkali-induced CNV model reduced the expression of pSTAT3 and the chemoattractant MCP-1, which corresponded with decreased macrophage infiltration, reduced VEGFA and MMP-2 and -9 expression, and improved corneal wound healing [86]. In addition, a crosstalk exists with VEGFA signaling, where the interaction of MMP14 with VEGFR1 modulates VEGFA bioavailability and signaling during CNV [56,87]. STAT activation upregulates specific MMPs (e.g., MMP-2, MMP-9, MMP-14), leading to endothelial cell activation, migration, and invasion [41,69]. Further investigations on the specific roles of various MMPs, particularly in HSK-induced CNV, are necessary to explore therapeutic options.

### 3.6. Rho/ROCK -MMPs Signaling

The regulation of endothelial cell migration and tube formation, a hallmark angiogenic process in which endothelial cells align and organize into lumen-containing vascular structures, is tightly controlled by the Rho-associated protein kinase (ROCK) pathway. This involves the regulation of actin-binding proteins like ezrin and moesin, thereby modulating endothelial cell shape, adhesion, and motility [51,88]. VEGFA signaling activates RhoA/ROCK to drive multiple aspects of angiogenesis, including endothelial permeability, migration, and survival, and pharmacological inhibition of ROCK has been shown to impair VEGFA-mediated angiogenic responses [89]. In addition, Sonic hedgehog (Shh) functions as an indirect angiogenic regulator and has been demonstrated to promote endothelial activation and neovascularization through ROCK-dependent signaling mechanisms [90,91]. While matrix metalloproteinases, such as MMP-9, and matricellular proteins, including osteopontin (OPN), are well-established downstream mediators of extracellular matrix remodeling during angiogenesis [90], their regulation within the context of Shh-ROCK signaling appears to be indirect and context-dependent rather than mechanistically linear. Collectively, these studies support a model in which VEGFA- and Shh-driven activation of Rho/ROCK signaling coordinates cytoskeletal reorganization and extracellular matrix remodeling to facilitate pathological angiogenesis

### 3.7. Notch-MMPs Signaling

Human corneal epithelial cells express multiple Notch receptors and ligands, implicating Notch signaling in the regulation of epithelial proliferation, differentiation, and wound repair at the ocular surface [51]. In endothelial cells, including HUVECs, Notch signaling has been shown to modulate VEGFA-driven angiogenic responses by regulating the expression and activity of matrix metalloproteinases involved in extracellular matrix remodeling. Specifically, activation of Notch signaling promotes the upregulation of MMP-2, MMP-9, and membrane-type MMP-14 in the context of VEGFA-induced angiogenesis, facilitating endothelial invasion and sprouting [62]. In vivo studies using Notch-ablated or Notch-inhibited models demonstrate reduced MMP-9 expression accompanied by attenuated neovascularization. Put together, these findings support a role for Notch signaling as a context-dependent modulator of MMP expression that integrates VEGFA cues with extracellular matrix remodeling during pathological angiogenesis.

### 3.8. MicroRNAs and JAK-STAT Signaling

MicroRNAs (miRNAs) are a class of short (~20–24 nucleotide) non-coding RNAs that regulate gene expression primarily by modulating target mRNA stability and translation. These small molecules perform regulatory functions in diverse developmental and biological events such as embryogenesis, organogenesis, differentiation, angiogenesis, and apoptosis, and they also play a crucial role in pathological processes [92,93,94]. MicroRNAs reported to be expressed in corneal tissues and implicated in corneal homeostasis or disease contexts include miR-31, miR-122, miR-126, miR-132, miR-146a, miR-184, miR-204/205, miR-451a, miR-673, and miR-706 [94]. Not all CNV-associated miRNAs converge directly on JAK–STAT signaling; many also regulate ERK pathways and extracellular matrix remodeling. However, these pathways intersect extensively with cytokine-driven STAT activation during inflammatory angiogenesis. Most studies examining miRNA regulation in CNV have focused on injury-induced models, such as chemical burn or suture-induced CNV. For example, miR-21 and miR-126 have been reported to be upregulated in chemical burn-induced CNV in albino rats, correlating with increased VEGFA expression, and have been associated with angiogenic progression. In contrast, miR-184 is downregulated, suggesting that these two sets of miRNA may play opposing roles in CNV [92]. Given that CNV is a major risk factor for corneal allograft rejection, a rat in vivo corneal transplantation model demonstrated that miR-673-5p exhibits anti-angiogenic properties, with JAK2 identified as a direct target. A binding site for miR-673-5p was observed on JAK2 mRNA, and the targeted inhibition and overexpression of miR-673-5p were found to promote and decrease phospho-JAK2 expression, respectively [95]. Reduced JAK2 phosphorylation following miR-673-5p overexpression was also associated with decreased IL-17 expression and Th17 differentiation, highlighting a role for miRNA-mediated JAK signaling in immunoinflammatory processes linked to CNV. Furthermore, in an extensive study of miRNA transcriptomics using a suture-induced CNV model, miR-21, miR-27a, miR-29, miR-142, and miR-1224 were, of note, differentially regulated in rats. Among these miRNAs, miR-21 displays context-dependent anti- and pro-angiogenic effects. miR-21 has been reported to suppress the expression of anti-MMP regulators such as TIMP3 in glioblastomas, and reducing miR-21 can elevate TIMP3 levels and dampen MMP activity, suggesting a pathway by which miR-21 may promote extracellular matrix remodeling, while in contrast, in injury-induced CNV, antagonism of miR-21 via antagomir treatment represses neovascularization and is associated with restoration of Sprouty2/4 expression and consequent attenuation of ERK phosphorylation [96,97]. Similarly, miR-1224 demonstrates context-dependent behavior, exerting either pro-angiogenic or anti-inflammatory effects depending on whether VEGFA induction or TNF-α suppression predominates [98,99,100]. In contrast, miR-27a, the miR-23-27 cluster, and miR-142 have been shown to exert predominantly pro-angiogenic effects in CNV [94].

In HSK-induced CNV, miR-132 expression has been shown to increase up to 20-fold, concomitant with increases in VEGFA and IL-17 levels. An in vivo inhibition and silencing of miR-132 reversed CNV and stromal lesions [93]. Furthermore, another study showed that overexpression of h-miR-146a or h-miR-424 in human corneal epithelium delayed wound healing by downregulating genes involved in cell proliferation and migration [91]. Given that efficient epithelial repair and barrier restoration are critical determinants of stromal inflammation and secondary angiogenic signaling following corneal injury, dysregulation of epithelial miRNAs may indirectly shape the angiogenic microenvironment during CNV. Another major miRNA with profound immunomodulatory consequences in HSK-induced CNV is miR-155. It has been found to be highly expressed in CNV by infiltrating CD45+, CD11b+, and F4/80+ macrophages, and its targeted inhibition reduced the incursion of inflammatory cells into the cornea and decreased corneal lesions [101]. In another study, miR-155 was shown to downregulate SOCS1, resulting in enhanced STAT3 signaling and altered PDCD4 expression, thereby promoting pro-inflammatory responses in atherosclerosis [102]. Interestingly, CNV-model dependent effect was likely observed in the bacterial-induced CNV model, where the opposite was found to be true of miR-155 [103].

The reasons underlying the seemingly contradictory roles of individual miRNAs within the same CNV model or across disease contexts remain poorly understood, further highlighting the complexity of miRNA-mediated regulation of CNV. One plausible explanation is that, unlike siRNAs, miRNAs typically target hundreds of transcripts with opposing functions, including both pro- and anti-angiogenic modulators. Consequently, miRNAs regulate signaling networks rather than individual genes, and their net effect is highly dependent on the prevailing signaling pathways active at the time of analysis. In addition, CNV is a dynamic pathological process that progresses through transient stages, from initiation to progression, each subject to negative feedback regulation. As a result, miRNA activity may exert apparently opposing effects when examined at different disease stages. Finally, miRNA actions are often highly cell-type specific, a crucial feature that is often obscured in studies relying on whole-cornea or bulk tissue analyses. While multiple miRNAs have been implicated in CNV, most mechanistic insights have been derived from sterile, injury-induced models. Whether these miRNA targets and signaling networks are preserved in HSV-1-induced CNV remains unresolved, particularly given the added complexity of immune modulation and chronic inflammatory signaling. Collectively, these findings highlight that microRNAs function as network-level modulators of CNV, exerting pro- or anti-angiogenic effects in a context-, cell-type-, and disease-stage-dependent manner. Further studies are therefore required to define miRNA-target interactions specifically within the context of herpetic stromal keratitis, rather than extrapolating from non-infection models.

To distinguish microRNAs directly implicated in HSK-induced CNV from those identified in other angiogenic settings, Table 3 summarizes microRNA classification across viral, bacterial, injury-induced, and non-corneal angiogenic models.

### 3.9. Hypoxia-HIF-1α Signaling

Hypoxia signaling, classically mediated by hypoxia-inducible factor-1α (HIF-1α), is a central regulatory axis in pathological angiogenesis and a well-established transcriptional driver of *VEGFA* expression. In the cornea, hypoxic stress can result from secondary consequences of epithelial injury, edema, stromal cellular infiltration, and metabolic rewiring during inflammation [104]. Low-oxygen conditions upregulate HIF-1α and VEGFA in human corneal epithelial cells, and a study using an injury-induced CNV model showed that HIF-1α expression increases in parallel with *VEGFA* upregulation and vessel ingrowth [105]. Similarly, another work leveraging oxygen-based approaches for the treatment of corneal hypoxia after alkali injury demonstrated persistent HIF-1α activation correlating with CNV and showed that reducing hypoxia was associated with reduced HIF-1α/VEGFA signals and achieved therapeutic outcomes [106].

Hypoxia-HIF-1α signaling remains comparatively underexplored in HSK-induced CNV, particularly relative to the well-characterized VEGFA-sVEGFR1 imbalance and immune-derived angiogenic cytokines. Nonetheless, pathological features associated with HSK-induced CNV make the HIF axis biologically plausible as a modulatory layer, as the inflamed corneal stroma becomes densely populated with infiltrating leukocytes and activated resident cells, leading to tissue remodeling, edema, and metabolic alterations that may favor HIF stabilization [107]. Therefore, HIF-1α may function less as an initiating driver and more as an amplifier that acts in parallel with other pathways that drive *VEGFA* induction in HSK already described, thereby contributing to disease severity. Given that inflammatory responses in HSK evolve in close temporal parallel with angiogenic expansion, investigation of hypoxia-HIF-1α signaling in HSK-induced CNV may uncover additional insight into the temporal regulation of *VEGFA* expression and help distinguish early inflammatory angiogenic signals from later stages of vessel persistence and maturation.

### 3.10. PI3K-AKT-mTOR Pathway in CNV

The phosphoinositide 3-kinase (PI3K)/AKT signaling pathway is a central regulator of epithelial homeostasis and stress responses in the cornea. In the corneal epithelium, growth factors such as epidermal growth factor (EGF), insulin-like growth factor-1 (IGF-1), insulin, and transforming growth factor-β (TGF-β) have been shown to activate PI3K/AKT signaling, thereby regulating epithelial proliferation, migration, survival, and wound healing. Activated PI3K then, in turn, phosphorylates AKT via PIP3, leading to downstream activation of signaling pathways involved in cell migration, proliferation, inflammation, and apoptosis, such as mTOR and GSK3β, with secondary effects on cellular redox homeostasis, including modulation of reactive oxygen species (ROS) levels [32]. PI3K-AKT-mTOR plays a crucial role in corneal epithelial cells’ homeostasis. Growth factors such as keratinocyte growth factor, hepatocyte growth factor (HGF), vascular endothelial growth factor B, nerve growth factor, and pigment epithelium-derived factor activate this pathway to mediate proliferation and wound healing in the corneal epithelium [32,108,109,110,111]. It is also an important signaling pathway target in tumor angiogenesis that is well studied. Studies that explore its role in CNV progression mostly do so in the context of injury-induced CNV and viral replication. A significant number of such studies focus primarily on the inhibition of members of this pathway as druggable targets or as part of drug repurposing or hit molecule inquiry. In one such study, Xanthatin, a sesquiterpene lactone with known anti-inflammatory properties, was shown to exhibit anti-CNV activity by inhibiting the expression of VEGFA, pVEGFR2, pSTAT3, and pAKT [112]. Similarly, studies have found that rapamycin, an mTOR inhibitor downstream of PI3K-AKT activation, reduced corneal inflammation, opacity, leukocyte invasion, and CNV [113,114,115,116]. ERK1/2 has also been implicated as another upstream signal regulating mTOR-mediated pathogenesis [113]. In a study focused on apoptosis and viral replication in HSV-1 mice and in vitro models, it was demonstrated that the upregulation of Serum and glucocorticoid-regulated protein kinase 1 (SGK1) and infection-induced apoptosis was reversed by treatment with PI3K inhibitor, LY294002, implicating the SGK1-PI3K-wnt signaling in disease progression and viral replication [117]. However, whether this axis affects VEGFA signaling in the HSK model remains unanswered.

Unlike the JAK-STAT pathway, which has an intrinsic paradoxical role in viral replication [31,118], the PI3K-AKT pathway supports host cell survival in normal physiological conditions, and its inhibition leads to apoptosis. Because many viruses rely on hijacking and activating this pathway to suppress apoptosis and prolong infected-cell survival, PI3K–AKT signaling has emerged as a complementary pathway that supports interferon-stimulated gene (ISG) expression, acting alongside canonical JAK-STAT signaling at the level of mRNA translation, promoting a sustained immune response [118,119,120]. Consequently, inhibition of this pathway can, in certain contexts, promote pro-viral outcomes by attenuating ISG expression [121]. Interestingly, antiviral small molecules such as prodigiosin, which has been shown to inhibit NF-κB and AKT-dependent prosurvival signaling, have promoted apoptosis in HSV-infected cells [122]. In contrast, BX795, which restricts ocular HSV-1 infection by inhibiting host AKT phosphorylation and associated stress-activated signaling pathways, has been shown to exhibit a therapeutic effect in mice [123,124]. Together, these findings underscore the dual role of PI3K-AKT signaling in host–virus interactions, where pathway activation may support antiviral interferon responses, yet also be exploited to sustain infected-cell survival during HSV infection. Interestingly, in an alkali-induced CNV model, p38 mitogen-activated protein kinase (MAPK) signaling has been linked to downstream activation of MAPK-activated protein kinase-2 (MK2), triggering the release of pro-inflammatory mediators and consequent leukocyte invasion [125,126]. Similarly, the ERK-MAPK and PI3K/AKT/mTOR pathways are both activated by VEGFA [127], suggesting crosstalk between PI3K-AKT-MAPK signaling pathways in CNV pathogenesis. Despite the role of PI3K-AKT-mTOR signaling in physiological and pathological angiogenesis, specific mechanistic details of its function in HSK-induced CNV are not fully established.

### 3.11. Heparanase in HSV-1 Pathology

Heparanase (HPSE) is an endoglycosidase and the only mammalian enzyme capable of cleaving heparan sulfate, a key extracellular matrix component that binds and sequesters numerous pro-angiogenic growth factors on cell surfaces and within the matrix. Through this activity, HPSE actively remodels the extracellular matrix and regulates the bioavailability of angiogenic signals, such as VEGFA [128], and therefore may contribute to pathological processes, including CNV. Given its role in modulating angiogenicity, HPSE is considered a potential therapeutic target in ocular neovascularization, particularly in Oxygen-induced retinopathy [129]. Furthermore, HPSE is a known driver of HSV-1 pathologies, and a study in our lab has established it as a driver of HSV-1 infection [123,129,130]. Therefore, here we will summarize current evidence of the role of HPSE in HSV-1 pathology, propose a model for its role in CNV, and highlight gaps in current mechanistic understanding.

### 3.12. Heparanase-AKT Signaling

HPSE has an established role in cancer, tumor progression, metastasis, and angiogenesis [131]. As previously explained, viruses depend on host mechanisms for survival and evade apoptosis by activating pathways such as the PI3K-AKT pathway. The role of HPSE in activating this pathway in pathologies like atherosclerosis, cancer metastasis, and angiogenesis has been widely studied, and it is known to activate this pathway to trigger endothelial cell migration and invasion [128,131,132,133]. In addition, scientists have speculated that HPSE may also mediate virus survival by activating this pathway [128]. Its known role in regulating AKT is of particular interest, given AKT’s role in mediating VEGFA signaling, as discussed earlier in this review. HPSE mediates PI3K/AKT activation via a mechanism that is not yet fully understood but is known to be independent of its enzymatic activity [128,133,134]. Interestingly, HPSE is known to regulate the signaling of a potent pro-angiogenic factor, FGF-2, via sustained PI3K/AKT pathway activation, and FGF-2 activation controls the downstream activation of other pro-angiogenic factors such as VEGFA and MMP-9 [135,136,137]. Furthermore, Koujah et al. showed that HSV-1-induced HPSE upregulation activates Wnt/β-catenin signaling via AKT activation to promote viral replication in vivo and in vitro [123]. Therefore, further investigation of whether HPSE-regulated β-catenin activation cross-talks with HPSE-activation of FGF-2 within the PI3K/AKT pathway will provide important insight into understanding its role in CNV.

### 3.13. Heparanase-VEGFA Signaling

As previously surmised in the literature, HPSE-induced *VEGFA* upregulation is mediated via a number of mechanisms, namely: non-enzymatic dependent upregulation of phospho-SFK; enzymatic action-dependent cleavage of heparan sulfate to release bound VEGFA; and syndecan-1 shedding induced VEGFR2 activation in endothelial cells [128,138]. In one study, five different CNV models: fungal infection, alkaline burn, suturing, immunogen implantation, and tumor cell implantation were used. Normal corneal tissues expressed HPSE in the Descemet’s membrane and the epithelium, and the upregulation of HPSE appeared consistent across the five experimental models, following assault. Increased HPSE expression was observed prior to the onset of neovascularization and correlated with the incursion of vessels into the cornea and with increased MMP-2 and -9 expression, decreasing with vessel regression, suggesting an association between HPSE activity, matrix remodeling, and angiogenic progression [47]. HPSE in HSK-induced CNV remains largely understudied; interestingly, a study from our laboratory demonstrated that cathepsin-L, a lysosomal protease, mediates HPSE-dependent upregulation of pro-angiogenic and inflammatory cytokines during HSV-1 infection [139]. Given the available evidence, current data suggest an important role for HPSE in HSK-induced CNV, and investigations that prioritize established mechanistic players in pathways that mediate this process are warranted.

**Figure 2:**
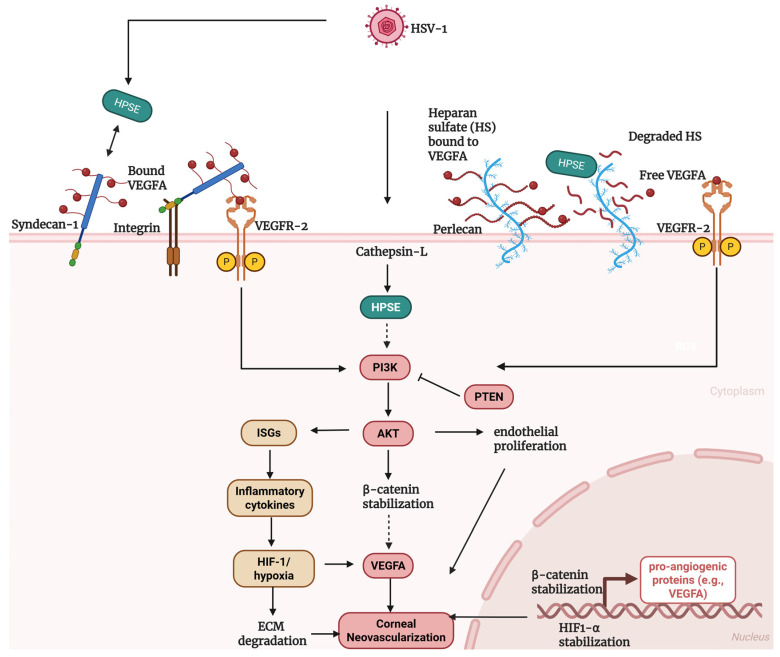
Proposed Mechanism of Heparanase-regulated angiogenic signaling in HSK-induced CNV. HSV-1 upregulates HPSE expression via cathepsin-L–dependent activation, and HPSE promotes syndecan-1 shedding indirectly by upregulating MMPs that cleave the syndecan-1 core protein, releasing a soluble ectodomain that retains heparan sulfate-bound VEGFA and increases its bioavailability for VEGFR2 activation. In parallel, HPSE directly cleaves heparan sulfate chains on syndecan-1, further liberating VEGFA from the cell surface and extracellular matrix. The shed syndecan-1-VEGFA complex can engage integrins on endothelial cells, facilitating VEGFR2 clustering and enhancing downstream angiogenic signaling. In addition, HPSE enzymatic activity cleaves heparan sulfate chains on extracellular matrix proteoglycans such as perlecan, releasing sequestered VEGFA into a freely diffusible pool capable of directly engaging VEGFR2 on endothelial cells. VEGFR2 activation converges on PI3K/AKT signaling, which is negatively regulated by PTEN. Activated AKT promotes endothelial proliferation and stabilizes β-catenin, driving transcriptional upregulation of pro-angiogenic genes, including VEGFA. Inflammatory cytokine induction and hypoxia-associated HIF-1α signaling further amplify VEGFA expression and extracellular matrix remodeling, collectively sustaining angiogenic signaling and promoting corneal neovascularization during HSV-1 infection. Solid arrows denote experimentally supported interactions, and dashed arrows indicate hypothesized or inferred signaling steps. Created in BioRender. Akinsiku, S. (2026) https://BioRender.com/lvwsjp7. Accessed on 27 January 2026.

Existing literature supports a multifaceted role for HPSE in HSV-1 pathogenesis and angiogenic signaling, while leaving key mechanistic questions unresolved, particularly in the context of CNV. To synthesize current evidence and explicitly highlight these gaps, we propose a working model in which HPSE integrates viral replication, host survival signaling, and pro-angiogenic remodeling during HSK-induced CNV (Figure 2).

### 3.14. Proposed Mechanism of HPSE-Regulation in HSK-Induced CNV

Following HSV-1 infection, pro-heparanase is proteolytically activated by cathepsin-L, generating signaling-competent HPSE within the corneal microenvironment. Notably, HPSE-driven AKT activation has been shown to stabilize β-catenin during HSV-1 infection, thereby reprogramming host transcriptional responses. We hypothesize that this AKT-β-catenin signaling axis contributes to the induction of pro-angiogenic gene expression, including VEGFA, within infected corneal epithelial cells, potentially in coordination with infection-induced inflammatory and hypoxic cues. In parallel, HPSE enzymatic activity promotes heparan sulfate cleavage and syndecan-1 shedding, increasing VEGFA bioavailability and enhancing VEGFR2 responsiveness in limbal endothelial cells. Together, these mechanisms position heparanase as both an initiator and an amplifier of PI3K/AKT-VEGFA signaling, driving endothelial activation and pathological CNV in HSK.

### 3.15. Osteopontin in Corneal Wound Healing

Osteopontin (OPN) is an extracellular matrix protein that is expressed in the corneal stroma, functioning in fibroblast proliferation, differentiation, and turnover, especially in corneal wound healing [140]. Its role in neovascularization and reversal of vascular calcification in cardiovascular tissues has also been established [141]. The loss of OPN was found to delay adhesion and migration in corneal fibroblasts in vitro, and in injury-induced corneal wound mouse models, impaired wound healing was observed in the corneal stroma of OPN KO mice compared to WT [140]. No significant difference was observed in wound healing in the corneal epithelium of mice, suggesting a specificity in ECM remodeling in the corneal stroma. OPN expression in the cornea tissues was also upregulated transiently following alkali-induced injury, and *VEGFA* mRNA expression was markedly increased in OPN KO compared to WT. However, these differences in *VEGFA* transcripts became less pronounced over time. In the context of HSV-1 infection, studies have shown that the loss of OPN ameliorates infection severity and corneal opacity, correlating with MMP-8 upregulation [140,142,143]. This upregulation has also been demonstrated in an alkali-induced injury model treated with dexamethasone, occurring in tandem with TGFB reduction [144,145]. Its role as an upstream regulator of immune response in HSK was also established, as lymph node cells from OPN^−/−^ mice expressed significantly higher anti-inflammatory cytokines, like IL-10, with diminished pro-inflammatory IL-12 expression [142]. Its effect on corneal opacity and inflammatory cytokines in HSK indirectly positions OPN as a negative regulator of CNV; however, its effects on angiogenic drivers in HSK, such as VEGFA, have yet to be investigated.

### 3.16. Syndecan-1 in CNV

Building on the multifaceted role of HPSE in VEGFA bioavailability and VEGFR2 activation described above, syndecan-1 represents a key downstream effector through which HPSE-mediated extracellular matrix remodeling may influence angiogenic signaling in CNV. In HSV-1 infection, HPSE cleaves heparan sulfate chains on syndecan-1 and promotes syndecan-1 ectodomain shedding indirectly by enhancing metalloproteinase activity, including MMP-3 and MMP-7. This supports the egress of viral particles from infected cells, thereby enhancing disease progression [146]. In parallel, syndecan-1 shedding alters the spatial presentation of heparan sulfate-bound growth factors, including VEGFA, thereby potentially modulating VEGFR2 activation in neighboring endothelial cells [138]. In addition, a study using an alkali-induced CNV model and syndecan-1 KO mice showed a 1.75-fold increase in neovascularization compared to WT mice, highlighting how its negative regulation of leukocyte-endothelial interactions might affect angiogenic and inflammatory responses [147]. These findings suggest that intact syndecan-1 may function as a regulatory checkpoint that limits excessive endothelial activation and vessel ingrowth following corneal injury. Given the role of syndecan-1 shedding in facilitating viral egress, available data suggest that syndecan-1 may function as a negative regulator of both viral spread and immunoinflammation-driven endothelial activation and migration in HSK-associated CNV. Whether or not this hypothesis holds true in the context of HSK-induced CNV remains to be determined.

## 4. Unanswered Questions and Future Directions

Studies in mouse models demonstrate that replication-competent HSV-1 rapidly induces VEGFA, followed by the growth of new blood vessels, while pharmacological or genetic inhibition of VEGFA signaling significantly reduces neovascularization and disease severity [58,148]. Notably, this induction may not simply reflect a generic host inflammatory response. Wuest et al. demonstrated that HSV-1 immediate-early regulatory proteins, particularly ICP4, directly transactivate the *VEGFA* promoter in infected corneal epithelial cells, and that this transcriptional activation requires cis-acting GC-box elements shared between the *VEGFA* promoter and HSV-1 early gene promoters [21]. These findings suggest that angiogenic signaling can be embedded within the viral transcriptional program through promoter-level hijacking of host genes. Similar VEGFA induction has been reported in infections with other non-oncogenic viruses, including dengue virus and hantaviruses [149], although the functional significance of this response remains unclear. It is therefore possible that *VEGFA* upregulation confers indirect advantages to viral fitness by remodeling the corneal microenvironment, affecting oxygen tension, nutrient availability, immune cell trafficking, and extracellular matrix composition in ways that are permissive to ongoing infection [149]. However, there is currently no consensus that VEGFA directly enhances viral spread or replication, and such effects have not been demonstrated. Future studies will determine whether VEGFA provides intrinsic value to viral replication or represents a byproduct of host transcriptional hijacking, an important unresolved question that remains to be answered.

An additional underexplored area in CNV research is the reciprocal effect of neovascularization on inflammatory signaling. Although CNV is commonly viewed as a pathological amplifier of inflammation, it remains possible that vascular stabilization introduces feedback mechanisms that partially restrain inflammatory signaling. Therefore, distinguishing between early angiogenic expansion and later vessel maturation in HSK may be critical for understanding how CNV dynamically shapes immunoinflammation-driven pathology during chronic infection.

As discussed in this review, pathway-level interventions targeting signaling nodes upstream or parallel to VEGFA can be effective therapeutic agents for HSK-induced CNV and should be explored. The JAK/STAT and PI3K/AKT/mTOR pathways are repeatedly implicated in CNV across injury-, inflammation-, and infection-induced models. These pathways regulate inflammatory gene transcription, cell survival, fibroblast activation, and endothelial responsiveness, positioning them as integrative hubs of pathological angiogenesis. Inhibiting such pathways may simultaneously reduce pro-angiogenic cytokine production, limit stromal activation, and blunt endothelial cell survival signals, which anti-VEGF therapy alone cannot achieve [113].

Collectively, available evidence suggests that targeted JAK/STAT modulation is a promising, but context-dependent, therapeutic strategy for HSK-induced CNV. In experimental corneal injury models, topical JAK inhibitors such as tofacitinib suppress inflammatory cell infiltration, reduce STAT1/STAT3 activation, and significantly attenuate corneal neovascularization, supporting the concept that JAK/STAT blockade can limit angiogenic amplification driven by inflammation and stromal activation [80]. More broadly, STAT3 signaling has been implicated as a central regulator of corneal inflammation, fibrosis, and angiogenesis across acquired corneal diseases, positioning this pathway as an integrative therapeutic target in CNV-prone settings [65]. However, JAK/STAT signaling also mediates interferon-driven antiviral immunity, and recent analyses emphasize its role as a “double-edged sword” in viral infections, raising the possibility that indiscriminate or poorly timed JAK inhibition during active HSV-1 replication could impair host defense or alter disease kinetics [150]. Together, these findings suggest that carefully timed, locally delivered, or cell-selective JAK/STAT-based interventions may offer translational benefit in HSK-associated CNV by suppressing pathological angiogenesis while minimizing disruption of antiviral immune control.

In addition, experimental studies demonstrate that MMP inhibition reduces CNV by preserving ECM integrity and preventing degradation of anti-angiogenic factors [64]. Rather than targeting angiogenic ligands directly, these approaches restrict the physical and biochemical conditions required for vessel ingrowth, creating a complementary anti-invasive mechanism that may be particularly relevant in chronic CNV. Gene-regulatory strategies, including microRNA-based interventions, further expand the therapeutic landscape. Specific microRNAs, such as miR-132, have been shown to amplify angiogenic signaling during ocular HSV infection by coordinating multiple downstream targets [93]. Targeting such microRNAs can suppress angiogenesis more broadly than single-ligand inhibition, while still allowing physiological wound-healing processes to proceed. 

Finally, emerging evidence suggests that host factors linking viral infection to tissue remodeling, such as HPSE, may represent novel therapeutic entry points. HPSE and syndecan-1 activity contribute to ECM degradation, growth-factor mobilization, and inflammatory signaling, all of which favor CNV progression. Although the precise mechanisms by which these factors integrate angiogenic and inflammatory signaling remain incompletely defined, their upstream positioning in multiple pathological processes makes them attractive candidates for adjunctive therapy. One class of such agents comprises heparan sulfate mimetics/HPSE inhibitors, which interfere with heparanase-mediated ECM degradation and release of bound pro-angiogenic ligands. Examples include PI-88 (muparfostat), a sulfated oligosaccharide that inhibits heparanase activity and has demonstrated anti-angiogenic effects in tumor angiogenesis models and early clinical oncology studies [151], as well as roneparstat (SST0001), a non-anticoagulant heparan sulfate mimetic that has shown anti-angiogenic and anti-tumor effects in preclinical myeloma models and has entered Phase I clinical evaluation [152]. In ocular disease contexts, PI-88 has also been shown to attenuate pathological vessel growth in experimental retinal neovascularization models, reducing both HPSE expression and VEGF signaling in oxygen-induced retinopathy mice [153]. Although these agents have not yet been specifically evaluated in HSK-induced CNV, their shared mechanism, limiting ECM remodeling and growth-factor mobilization, aligns with key pathological features of CNV associated with HSK.

Future therapeutic paradigms are therefore likely to integrate VEGFA inhibition with pathway-level modulation, anti-remodeling strategies, and gene-regulatory interventions to achieve more durable and disease-modifying control of CNV.

## 5. Conclusions

VEGFA is a central driver of CNV, and anti-VEGF therapies have transformed the management of pathological angiogenesis in the eye [148]. However, both experimental and clinical evidence increasingly demonstrate that VEGFA inhibition alone is insufficient for durable CNV control [148,154,155], particularly in inflammatory and infection-driven contexts such as HSK. CNV in these settings is not merely the result of excess VEGFA signaling, but rather reflects a multifactorial disease state sustained by chronic inflammation, ECM remodeling, and dysregulated host–pathogen interactions. These limitations necessitate therapeutic strategies that extend beyond VEGFA blockade. One major limitation of anti-VEGF monotherapy is its inability to address upstream inflammatory drivers of angiogenesis [42]. While VEGFA neutralization can suppress endothelial sprouting, it does not adequately resolve the inflammatory signaling networks that continue to fuel angiogenic pressure [148]. This helps explain why CNV often recurs after cessation of therapy and why established vessels may persist despite VEGFA inhibition [156]. A second limitation lies in the loss of endogenous anti-angiogenic control mechanisms, which anti-VEGF agents do not restore, such as sVEGFR1, allowing vessels to invade and stabilize even when VEGF signaling is partially suppressed [28].

In summary, CNV, particularly in inflammatory and infectious diseases like HSK, cannot be fully explained or controlled by VEGFA signaling alone. Anti-VEGF therapy remains an essential component of treatment, but its limitations highlight the need for combination and multi-target approaches that address inflammation, stromal remodeling, and angiogenic amplification. In this review, we have synthesized evidence supporting pathway-level regulation of CNV from multiple experimental models and integrated these findings within the context of HSK-induced CNV. We highlighted underexplored regulators, including heparanase, as upstream integrators of viral replication, extracellular matrix remodeling, and pro-angiogenic signaling that shape immune-driven pathology. This pathway-level perspective emphasizes therapeutic opportunities that target angiogenic and stromal amplification mechanisms upstream of immune effector responses.

## Figures and Tables

**Table 1:** Comparison of Features of Angiogenesis in Normal Tissues and Corneal Neovascularization (CNV).

Feature	Physiological Angiogenesis	CNV
Tissue context	Vascularized tissues	Avascular, immune-privileged cornea
Primary trigger	Development, growth, tissue repair	Chronic inflammation, infection (e.g., HSV-1)
Role of hypoxia	Context-dependent or transient	Secondary, persistent, or modulatory
VEGF regulation	Tightly controlled	Sustained, dysregulated expression
Anti-angiogenic control	Preserved	Compromised (loss of sVEGFR-1 and TIMPs)
Inflammatory input	Context-dependent, resolving	Dominant and chronic
Cellular drivers	Endothelial-centric	Multicellular (epithelium, stroma, immune)
Vessel architecture	Organized and functional	Disorganized, leaky vessels

**Table 2:** Distinct roles of corneal resident and infiltrating cell populations in HSK-induced CNV.

Corneal Cell Type	Major Angiogenic Outputs	Functional Role in CNV
Corneal epithelial cells	VEGFA, IL-6, pro-inflammatory cytokines	Initiation of angiogenic signaling
Stromal/keratocytes	MMP-2, MMP-9, ECM remodeling	Amplification and stromal invasion
Limbal vascular endothelial cells	Endothelial migration, tube formation	Execution of neovascular growth
Immune cells (CD4^+^T cells, macrophages, neutrophils)	VEGFA, IL-17, TNF-α	Modulation and inflammatory amplification
Corneal endothelial cells	Barrier dysfunction, metabolic stress	Permissive role in CNV progression
Pericytes/vascular support cells	Vessel stabilization and persistence	Maintenance and maturation of CNV

**Table 3:** Reviewed microRNAs implicated across CNV disease context.

Disease Context	Pro-Angiogenic miRNAs	Anti-Angiogenic miRNAs	Unresolved/Context-Dependent miRNAs
HSk-induced CNV	miR-132		miR-155
Bacterial-induced CNV		miR-155	
Injury-induced CNV (alkali burn, suture, and transplant)	miR-21;	miR-184	miR-21;
	miR-126;	miR-673-5p;	miR-122;
	miR-27a;		miR-1224
	miR-23/27 cluster miR-142		
Other angiogenic models (non-corneal)	miR-21;	miR-184	miR-155;
	miR-126; miR-27a		miR-1224

## Data Availability

No new data were created or analyzed in this study.

## Abbreviations

The following abbreviations are used in this manuscript:

AKT	Protein kinase B
Ang-2	Angiopoietin-2
AP-1/AP-2	Activator protein-1/Activator protein-2
BBB	Blood–brain barrier
CNV	Corneal neovascularization
COX-2	Cyclooxygenase-2
CNTF	Ciliary neurotrophic factor
CXCL	C-X-C motif chemokine ligand
CCL	C-C motif chemokine ligand
ECM	Extracellular matrix
EGF	Epidermal growth factor
ERK	Extracellular signal-regulated kinase
FGF2	Fibroblast growth factor-2
FLT-1	Fms-like tyrosine kinase-1 (VEGFR1)
GSK3β	Glycogen synthase kinase-3 beta
HGF	Hepatocyte growth factor
HIF-1α	Hypoxia-inducible factor-1 alpha
HPSE	Heparanase
HS	Heparan sulfate
HSK	Herpes simplex keratitis
HSV-1	Herpes simplex virus type-1
HUVECs	Human umbilical vein endothelial cells
ICP4	Infected cell protein-4
IFN	Interferon
IFNAR	Interferon-alpha receptor
IL	Interleukin
ISGs	Interferon-stimulated genes
JAK	Janus kinase
KDR	Kinase insert domain receptor (VEGFR2)
LIF	Leukemia inhibitory factor
MAPK	Mitogen-activated protein kinase
MCP-1	Monocyte chemoattractant protein-1
miRNA/miR	MicroRNA
MK2	MAPK-activated protein kinase-2
MMP	Matrix metalloproteinase
mTOR	Mechanistic target of rapamycin
NF-κB	Nuclear factor kappa-B
NO	Nitric oxide
NR-1	Neuregulin-1
OPN	Osteopontin
OSM	Oncostatin M
PDCD4	Programmed cell death protein-4
PDGF	Platelet-derived growth factor
PEDF	Pigment epithelium-derived factor
PI3K	Phosphatoinositide 3-kinase
PIAS	Protein inhibitor of activated STAT
PMN	Polymorphonuclear neutrophils
PTEN	Phosphatase and tensin homolog
PTP	Protein tyrosine phosphatase
Rho/ROCK	Rho-associated protein kinase
ROS	Reactive oxygen species
SFK/phosphoSFK	Src family kinases
SGK1	Serum and glucocorticoid-regulated kinase-1
Shh	Sonic hedgehog
sVEGFR1	Soluble vascular endothelial growth factor receptor-1
SOCS	Suppressor of cytokine signaling
STAT	Signal transducer and activator of transcription
TGFB/TGF-β	Transforming growth factor-beta
Th1	T helper type-1
Th17	T helper type-17
TIMP	Tissue inhibitor of metalloproteinases
TNF-α	Tumor necrosis factor-alpha
TSP-1/TSP-2	Thrombospondin-1/Thrombospondin-2
UL	Unique long (HSV gene region)
VEGFA	Vascular endothelial growth factor-A
VEGFR1/VEGFR2	Vascular endothelial growth factor receptor-1/-2
VECs	Vascular endothelial cells
WT	Wild type
